# *catena*-Poly[[di­aqua­bis­(1,3-di­hydro-3-oxo­isobenzo­furan-1-acetato)­copper(II)]-μ-*N*,*N*′-(ethane-1,2-di­yl)dinicotinamide]

**DOI:** 10.1107/S2414314623007642

**Published:** 2023-09-08

**Authors:** Sean R. Pumford, Robert L. LaDuca

**Affiliations:** a H. H. Dow High School, Midland, MI 48640, USA; b E-35 Holmes Hall, Michigan State University, Lyman Briggs College, 919 E. Shaw Lane, East Lansing, MI 48825, USA; Purdue University, USA

**Keywords:** crystal structure, coordination polymer, *in situ* lactonization, dibf, edn

## Abstract

The title compound, {[Cu(C_10_H_7_O_4_)_2_(C_14_H_14_N_4_O_2_)(H_2_O)_2_]_*n*_, contains octa­hed­rally coordinated Cu^II^ ions ligated by two bis­(1,3-di­hydro-3-oxo-1-isobenzo­furan­acetate (dibf) ligands and two *trans* water mol­ecules, linked by *N*,*N*′-(ethane-1,2-di­yl)dinicotinamide (edn) ligands into mono-periodic coordination polymer chains. The dibf ligands exhibit a pseudo-mirror positional disorder over two positions in a 89.2 (3)/10.8 (3) ratio; the central amide groups of the edn ligands are disordered pseudo-rotationally in the same ratio. These mono-periodic chain motifs are held into supra­molecular di-periodic supra­molecular layers by means of N—H⋯O hydrogen bonding between edn amide groups and unligated dibf carboxyl­ate O atoms. In turn, the supra­molecular layers are held by crystal packing forces into the full crystal structure of the title compound.

## Abstract 2

*In situ* lactonization of 2-carb­oxy­cinnamic acid to form bis­(1,3-di­hydro-3-oxo-1-isobenzo­furan­acetate (dibf) resulted in the the crystallization of [Cu(dibf)(edn)(H_2_O)_2_]_*n*_ coordination polymer chains [edn = *N*,*N*′-(ethane-1,2-di­yl)dinicotinamide].

## Graphical abstract

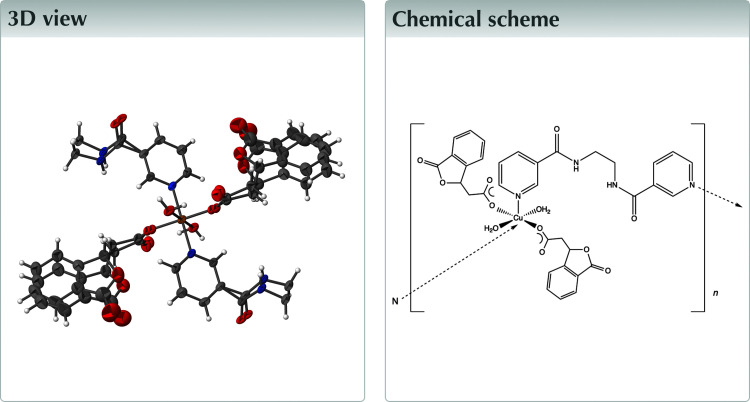

## Structure description

Our group (Przybyla *et al.*, 2019 ▸) and other groups (Wang *et al.*, 2013 ▸) have demonstrated the utility of *N*,*N*′-(ethane-1,2-di­yl)dinicotinamide) (edn) for the construction of divalent metal coordination polymers. The title complex was obtained by hydro­thermal reaction of copper nitrate, 2-carb­oxy­cinnamic acid, and edn under basic conditions.

**Figure 1:**
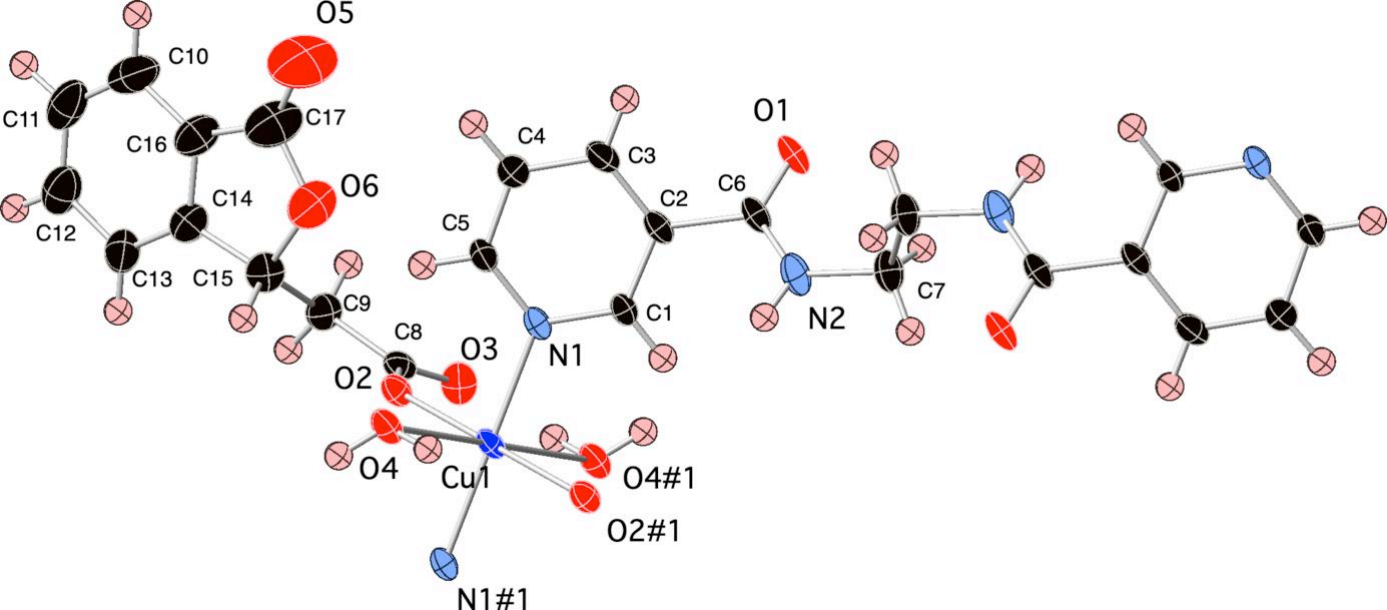
Copper coordination environment in the title compound with full ligand set. Displacement ellipsoids are drawn at the 50% probability level. The minor disorder components are not shown. Color code: Co, dark blue; O, red; N, light blue; C, black; H, pink. Symmetry codes are as listed in Table 1 ▸.

**Figure 2:**
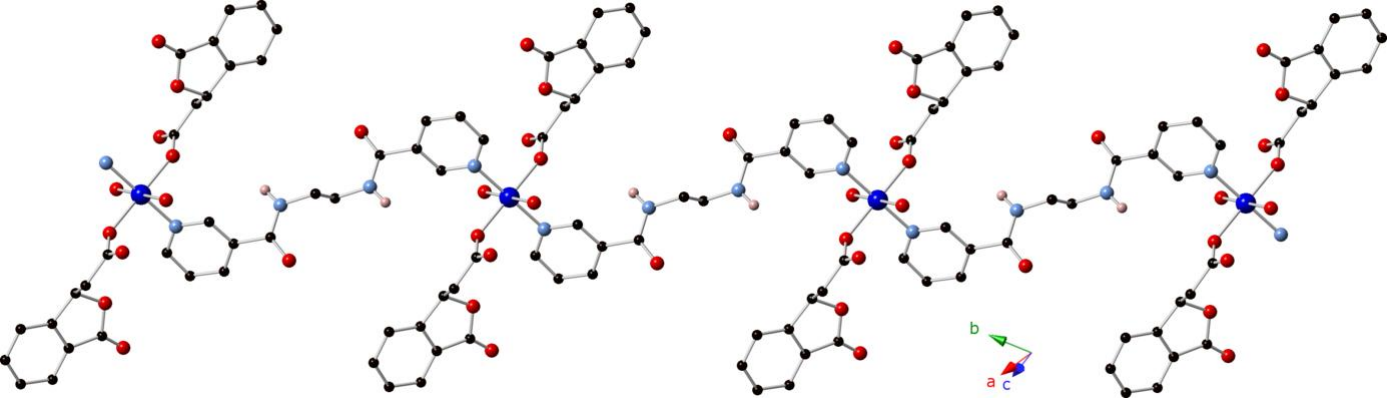
[Cu(dibf)(edn)(H_2_O)_2_]_*n*_ coordination polymer chain in the title compound.

**Figure 3:**
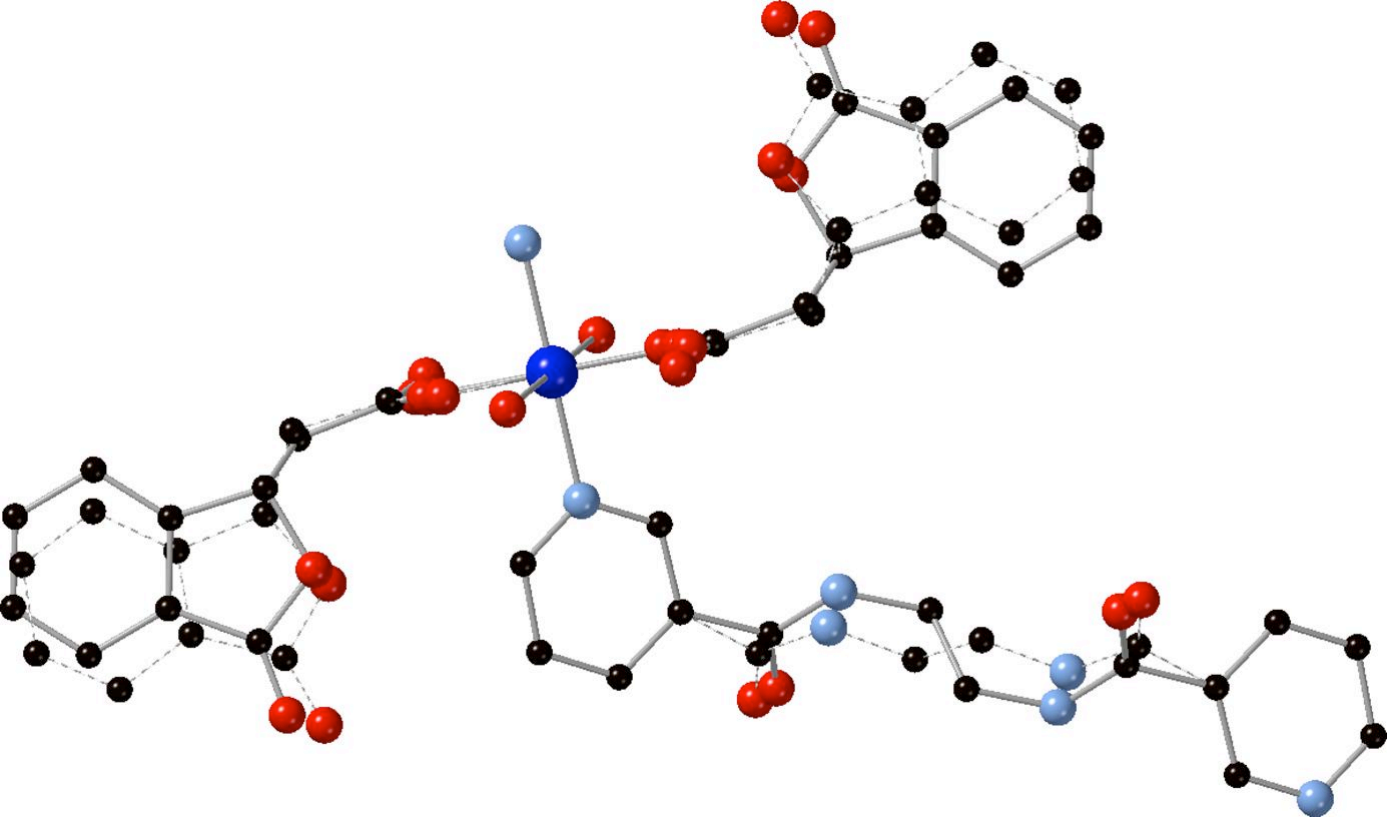
Copper coordination environment in the title compound with full ligand set showing major and minor disordered components. Color code: Co, dark blue; O, red; N, light blue; C, black. H atoms have been omitted. The minor disordered components have bonds drawn as dashed lines.

The asymmetric unit of the title compound contains a divalent copper atom on a crystallographic inversion center, one bis(1,3-dihydro-3-oxo-1-isobenzofuranacetate (dibf) ligand generated from the *in situ* lactonization of 2-carb­oxy­cinnamic acid (Murray & LaDuca, 2014 ▸), one weakly bound water mol­ecule, and half of an edn ligand whose central C—C σ bond is sited over another crystallographic inversion center. Operation of the inversion center at the Cu^II^ atom results in a Jahn–Teller-distorted {N_2_O_4_} coordination environment (Fig. 1 ▸) whose elongated axial positions are filled by the bound water mol­ecules. *Trans* pyridyl N donor atoms from two edn ligands, and *trans* carboxyl­ate O atoms from two dibf ligands occupy the four equatorial positions. Bond lengths and angles within the coordination sphere are listed in Table 1 ▸. The dibf ligands in the title complex serve as monodentate capping ligands. Neighboring copper atoms are linked by dipodal edn ligands to construct [Cu(dibf)(edn)(H_2_O)_2_]_*n*_ coordination polymer chains that are oriented along the [110] direction (Fig. 2 ▸). Both the edn as well as the dibf ligands in the title complex are disordered (Fig. 3 ▸). For the edn ligand, the central *N*,*N*′-(ethane-1,2-di­yl)di­amide unit is disordered by a pseudo-rotation around the center of the ethyl­ene group. Both the major and minor moiety are located on the crystallographic inversion center and are both exactly inversion symmetric. The dibf disorder involves a pseudo-mirror operation, with inverted handedness for the saturated carbon atom C15 of the isobenzo­furan­one. The disorder is correlated *via* a close contact between hydrogen atoms of the major moiety edn ligand and the minor moiety dibf ligand [H7*B*⋯H10*B* ^i^ = 1.72 Å, C7⋯C10*B* ^i^ = 3.31 (2) Å; symmetry code: (i) −*x*, 1 − *y*, −1 − *z*]. The disorder ratio in both ligands refined to exactly identical values, 89.2 (3)/10.8 (3), indicating that the disorder of the edn ligand causes the disorder of the dibf ligand. The minor moieties of the dibf ligand are incompatible with each other due to a close contact between the lactone oxygen atoms O5*B* [O5*B*⋯O5*B* ^ii^ = 2.91 (7) Å; symmetry code: (ii) −*x*, 2 − *y*, −1 − *z*].

**Figure 4:**
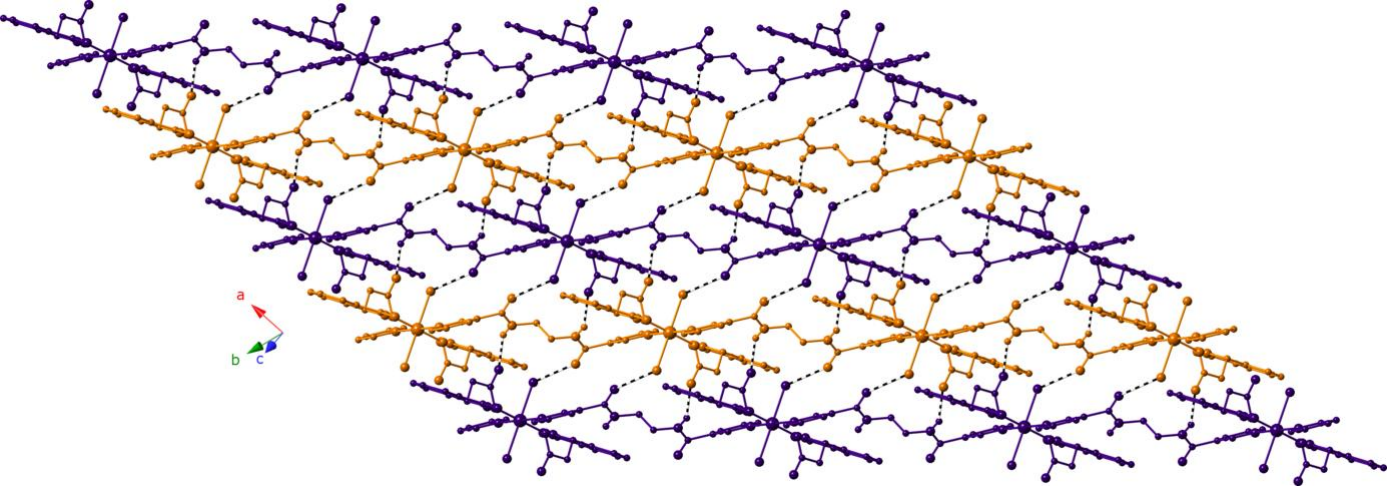
Supra­molecular layer of [Cu(dibf)(edn)(H_2_O)_2_]_*n*_ chains in the title compound.

**Figure 5:**
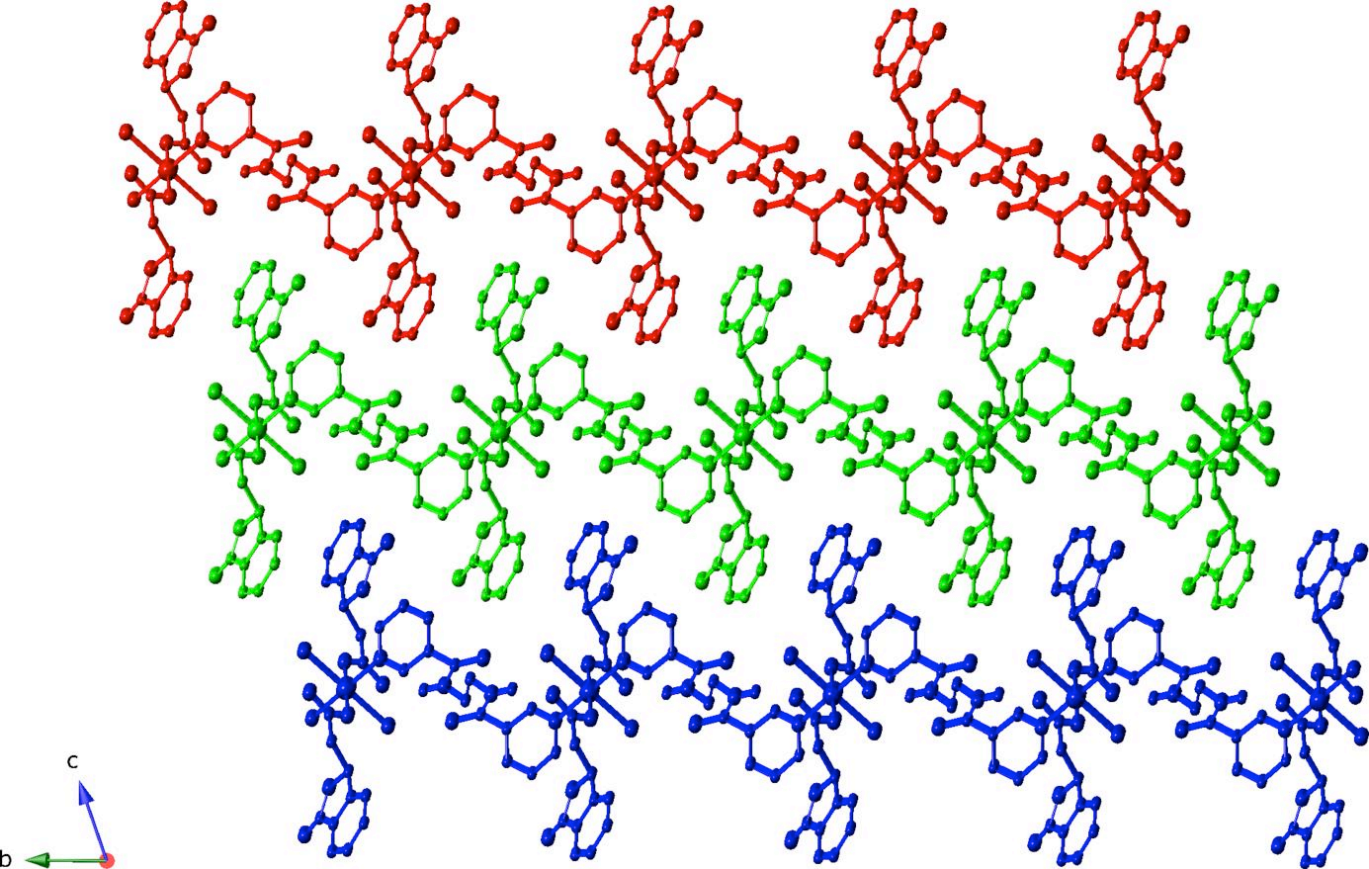
Stacking of supra­molecular layers in the title compound.

The [Cu(dibf)(edn)(H_2_O)_2_]_*n*_ chains aggregate into supra­molecular layers parallel to the *ab* crystal planes (Fig. 4 ▸) by hydrogen-bonding donation from edn amide N—H groups to unligated dibf carboxyl­ate O atoms, and by hydrogen-bonding donation from bound water mol­ecules to ebn amide C=O carbonyl groups (Table 2 ▸). Crystal packing forces between adjacent supra­molecular layers along the *c*-axis direction afford the full tri-periodic crystal structure of the title compound (Fig. 5 ▸).

## Synthesis and crystallization

Cu(NO_3_)_2_·2.5H_2_O (86 mg, 0.37 mmol), 2-carb­oxy­cinnamic acid (ccaH_2_) (72 mg, 0.37 mmol), *N*,*N*′-(ethane-1,2-di­yl)dinicotinamide (edn) (99 mg, 0.37 mmol), and 0.75 ml of a 1.0 *M* NaOH solution were placed into 10 ml of distilled water in a Teflon-lined acid digestion bomb. The bomb was sealed and heated in an oven at 393 K for 24 h, and then cooled slowly to 273 K. Green crystals of the title complex were obtained in 19% yield. Analysis calculated for C_34_H_32_CuN_4_O_12_: C, 54.29; H, 4.29; N, 7.45%. Found: C, 54.01; H, 4.62; N, 7.11%

## Refinement

Crystal data, data collection and structure refinement details are summarized in Table 3 ▸. All H atoms attached to C atoms were placed in calculated positions and refined with a riding model, with the H atoms attached to N or O found *via* difference map and then restrained (with the exception of the minor disorder N—H bond in the dibf ligand (see below). The dibf carboxyl­ate ligands and the amide groups of the edn ligands were refined as disordered over two sets of positions in a 89.2 (3)/10.8 (3) ratio. The dibf ligand exhibits a pseudo-mirror positional disorder; the edn amide group displays a pseudo-rotational relationship between its disordered components. These were treated with PART commands. Within the disordered components, SIMU commands were employed to restrain the *U_ij_* components of the atomic displacement parameters in order to avoid non-positive def­inite atomic displacement parameters. SADI and SAME commands were employed for the disordered components to restrain the bond lenghts and angles of major and minor moieties to be the same within an e.s.d. of 0.02 Å, to ensure chemically reasonable bond length and angle values. The H atoms belonging to the bound water mol­ecules were restrained with a DFIX command at 0.84 (2) Å. The amide proton of the major component of the disordered edn ligand was found and had its N—H bond distance restrained with a DFIX command at 0.88 (2) Å. The amide proton of the minor component was placed geometrically. EADP commands were used to constrain the atomic displacement parameters for carboxyl­ate major and minor disordered components of the dibf ligand to exactly the same values, again to avoid non-positive definite atomic displacement parameters.

## Supplementary Material

Crystal structure: contains datablock(s) I, 1R. DOI: 10.1107/S2414314623007642/zl4058sup1.cif

Structure factors: contains datablock(s) I. DOI: 10.1107/S2414314623007642/zl4058Isup3.hkl

CCDC reference: 1492694

Additional supporting information:  crystallographic information; 3D view; checkCIF report

## Figures and Tables

**Table 1:** Selected geometric parameters (Å, °)

Cu1—O2	2.008 (3)	Cu1—N1	2.0146 (16)
Cu1—O4	2.4790 (17)		
			
O2—Cu1—O4	94.84 (13)	O2*B*—Cu1—O4	98.1 (14)
O2—Cu1—O4^i^	85.16 (13)	O2*B*—Cu1—N1	91.3 (16)
O2—Cu1—N1^i^	89.68 (15)	O2*B*—Cu1—N1^i^	88.7 (16)
O2—Cu1—N1	90.32 (15)	N1—Cu1—O4^i^	91.36 (6)
O2*B* ^i^—Cu1—O4	81.9 (14)	N1—Cu1—O4	88.64 (6)

Symmetry code: (i) 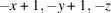 .

**Table 2:** Hydrogen-bond geometry (Å, °)

*D*—H⋯*A*	*D*—H	H⋯*A*	*D*⋯*A*	*D*—H⋯*A*
N2—H2⋯O3^ii^	0.85 (2)	1.98 (2)	2.801 (4)	163 (3)
O4—H4*A*⋯O3	0.86 (2)	1.83 (2)	2.674 (4)	170 (3)
O4—H4*B*⋯O1^iii^	0.81 (2)	2.05 (2)	2.860 (3)	171 (3)
C1—H1⋯O2	0.95	2.52	2.987 (5)	111

Symmetry codes: (ii) 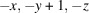 ; (iii) 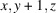 .

**Table 3:** Experimental details

Crystal data	
Chemical formula	[Cu(C_10_H_7_O_4_)_2_(C_14_H_14_N_4_O_2_)(H_2_O)_2_]
*M*_r_	752.17
Crystal system, space group	Triclinic, *P* 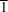
Temperature (K)	173
*a*, *b*, *c* (Å)	7.9413 (13), 10.4614 (16), 11.3198 (18)
α, β, γ (°)	70.6534 (18), 87.8784 (19), 73.9621 (18)
*V* (Å^3^)	851.2 (2)
*Z*	1
Radiation type	Mo *K*α
μ (mm^−1^)	0.71
Crystal size (mm)	0.61 × 0.31 × 0.25

Data collection	
Diffractometer	Bruker APEXII CCD
Absorption correction	Multi-scan (*SADABS*; Krause *et al.*, 2015 ▸)
*T*_min_, *T*_max_	0.682, 0.745
No. of measured, independent and observed [*I* > 2σ(*I*)] reflections	14134, 3137, 2849
*R*_int_	0.031
(sin θ/λ)_max_ (Å^−1^)	0.603

Refinement	
*R*[*F* ^2^ > 2σ(*F* ^2^)], *wR*(*F* ^2^), *S*	0.036, 0.093, 1.06
No. of reflections	3137
No. of parameters	381
No. of restraints	591
H-atom treatment	H atoms treated by a mixture of independent and constrained refinement
Δρ_max_, Δρ_min_ (e Å^−3^)	0.45, −0.18

Computer programs: *COSMO* (Bruker, 2009 ▸), *SAINT* (Bruker, 2014 ▸), *SHELXS* (Sheldrick, 2008 ▸), *SHELXL2018/3* (Sheldrick, 2015 ▸), *OLEX2* (Dolomanov *et al.*, 2009 ▸), and *CrystalMaker X* (Palmer, 2020 ▸).

## full crystallographic data

### Crystal data

**Table:**

[Cu(C_10_H_7_O_4_)_2_(C_14_H_14_N_4_O_2_)(H_2_O)_2_]	*Z* = 1
*M**_r_* = 752.17	*F*(000) = 389
Triclinic, *P*1	*D*_x_ = 1.467 Mg m^−^^3^
*a* = 7.9413 (13) Å	Mo *K*α radiation, λ = 0.71073 Å
*b* = 10.4614 (16) Å	Cell parameters from 8423 reflections
*c* = 11.3198 (18) Å	θ = 2.4–25.3°
α = 70.6534 (18)°	µ = 0.71 mm^−^^1^
β = 87.8784 (19)°	*T* = 173 K
γ = 73.9621 (18)°	Block, green
*V* = 851.2 (2) Å^3^	0.61 × 0.31 × 0.25 mm

### Data collection

**Table:**

Bruker APEXII CCD diffractometer	2849 reflections with *I* > 2σ(*I*)
φ and ω scans	*R*_int_ = 0.031
Absorption correction: multi-scan (SADABS; Krause *et al.*, 2015)	θ_max_ = 25.4°, θ_min_ = 1.9°
*T*_min_ = 0.682, *T*_max_ = 0.745	*h* = −9→9
14134 measured reflections	*k* = −12→12
3137 independent reflections	*l* = −13→13

### Refinement

**Table:**

Refinement on *F*^2^	591 restraints
Least-squares matrix: full	Hydrogen site location: mixed
*R*[*F*^2^ > 2σ(*F*^2^)] = 0.036	H atoms treated by a mixture of independent and constrained refinement
*wR*(*F*^2^) = 0.093	*w* = 1/[σ^2^(*F*_o_^2^) + (0.0507*P*)^2^ + 0.4221*P*] where *P* = (*F*_o_^2^ + 2*F*_c_^2^)/3
*S* = 1.06	(Δ/σ)_max_ = 0.001
3137 reflections	Δρ_max_ = 0.45 e Å^−^^3^
381 parameters	Δρ_min_ = −0.18 e Å^−^^3^

### Special details

**Table:**

Geometry. All esds (except the esd in the dihedral angle between two l.s. planes) are estimated using the full covariance matrix. The cell esds are taken into account individually in the estimation of esds in distances, angles and torsion angles; correlations between esds in cell parameters are only used when they are defined by crystal symmetry. An approximate (isotropic) treatment of cell esds is used for estimating esds involving l.s. planes.

### Fractional atomic coordinates and isotropic or equivalent isotropic displacement parameters (Å^2^)

**Table:**

	*x*	*y*	*z*	*U*_iso_*/*U*_eq_	Occ. (<1)
Cu1	0.500000	0.500000	0.000000	0.02846 (14)	
O1	0.3728 (4)	−0.1070 (3)	0.1206 (3)	0.0390 (6)	0.892 (7)
N2	0.1654 (3)	0.0933 (3)	0.0117 (3)	0.0330 (6)	0.892 (7)
H2	0.111 (3)	0.175 (2)	0.013 (3)	0.040*	0.892 (7)
C6	0.3054 (5)	0.0207 (3)	0.0920 (4)	0.0286 (8)	0.892 (7)
C7	0.0677 (3)	0.0261 (3)	−0.0433 (3)	0.0346 (7)	0.892 (7)
H7A	0.150294	−0.054618	−0.061092	0.041*	0.892 (7)
H7B	0.007836	0.094328	−0.123817	0.041*	0.892 (7)
O1B	0.418 (4)	−0.129 (3)	0.143 (3)	0.0390 (6)	0.108 (7)
N2B	0.171 (3)	0.054 (2)	0.060 (2)	0.031 (3)	0.108 (7)
H2B	0.116521	0.143623	0.046657	0.037*	0.108 (7)
C6B	0.330 (4)	−0.008 (2)	0.118 (4)	0.032 (3)	0.108 (7)
C7B	0.093 (3)	−0.039 (3)	0.019 (3)	0.0346 (7)	0.108 (7)
H7BA	0.103073	−0.128176	0.088782	0.041*	0.108 (7)
H7BB	0.155379	−0.060916	−0.052245	0.041*	0.108 (7)
O2	0.2836 (3)	0.5325 (5)	−0.1046 (4)	0.0294 (6)	0.892 (3)
O3	0.0763 (4)	0.6549 (4)	−0.0126 (2)	0.0420 (7)	0.892 (3)
O5	0.1617 (6)	0.8738 (5)	−0.5706 (3)	0.1142 (15)	0.892 (3)
O6	0.1684 (3)	0.7086 (3)	−0.3839 (2)	0.0621 (7)	0.892 (3)
C8	0.1300 (4)	0.6075 (4)	−0.0992 (3)	0.0303 (8)	0.892 (3)
C9	−0.0022 (3)	0.6365 (3)	−0.2048 (2)	0.0354 (6)	0.892 (3)
H9A	−0.083854	0.578265	−0.172123	0.043*	0.892 (3)
H9B	−0.071910	0.736660	−0.229007	0.043*	0.892 (3)
C10	−0.1702 (6)	0.7882 (5)	−0.6291 (3)	0.0662 (10)	0.892 (3)
H10	−0.160421	0.863867	−0.701383	0.079*	0.892 (3)
C11	−0.2980 (5)	0.7229 (5)	−0.6247 (4)	0.0636 (10)	0.892 (3)
H11	−0.377078	0.751837	−0.695701	0.076*	0.892 (3)
C12	−0.3140 (5)	0.6154 (4)	−0.5186 (4)	0.0587 (9)	0.892 (3)
H12	−0.405421	0.572626	−0.517416	0.070*	0.892 (3)
C13	−0.2001 (4)	0.5681 (4)	−0.4133 (3)	0.0475 (7)	0.892 (3)
H13	−0.212374	0.494276	−0.340170	0.057*	0.892 (3)
C14	−0.0681 (3)	0.6322 (3)	−0.4186 (2)	0.0394 (6)	0.892 (3)
C15	0.0716 (3)	0.6085 (3)	−0.3211 (3)	0.0401 (6)	0.892 (3)
H15	0.150486	0.510055	−0.298408	0.048*	0.892 (3)
C16	−0.0538 (5)	0.7401 (4)	−0.5237 (3)	0.0528 (8)	0.892 (3)
C17	0.0976 (5)	0.7872 (5)	−0.5028 (4)	0.0710 (10)	0.892 (3)
O2B	0.320 (4)	0.526 (5)	−0.097 (4)	0.0294 (6)	0.108 (3)
O3B	0.083 (5)	0.664 (4)	−0.050 (3)	0.0420 (7)	0.108 (3)
O5B	0.166 (4)	0.930 (3)	−0.539 (3)	0.090 (7)	0.108 (3)
O6B	0.094 (2)	0.798 (2)	−0.3578 (14)	0.059 (3)	0.108 (3)
C8B	0.156 (4)	0.583 (4)	−0.110 (3)	0.032 (3)	0.108 (3)
C9B	0.052 (3)	0.580 (2)	−0.2211 (19)	0.035 (3)	0.108 (3)
H9BA	0.133316	0.523589	−0.265596	0.041*	0.108 (3)
H9BB	−0.038327	0.530667	−0.187187	0.041*	0.108 (3)
C10B	−0.101 (4)	0.822 (3)	−0.653 (2)	0.062 (4)	0.108 (3)
H10B	−0.057226	0.881041	−0.722680	0.074*	0.108 (3)
C11B	−0.230 (4)	0.764 (3)	−0.669 (2)	0.061 (4)	0.108 (3)
H11B	−0.277053	0.785706	−0.751247	0.073*	0.108 (3)
C12B	−0.294 (5)	0.674 (4)	−0.569 (2)	0.056 (3)	0.108 (3)
H12B	−0.376333	0.630000	−0.585168	0.067*	0.108 (3)
C13B	−0.237 (3)	0.649 (3)	−0.447 (2)	0.053 (3)	0.108 (3)
H13B	−0.280825	0.590247	−0.376423	0.064*	0.108 (3)
C14B	−0.112 (3)	0.715 (3)	−0.4326 (16)	0.046 (3)	0.108 (3)
C15B	−0.036 (2)	0.721 (2)	−0.3141 (15)	0.044 (3)	0.108 (3)
H15B	−0.130897	0.774886	−0.273811	0.053*	0.108 (3)
C16B	−0.038 (4)	0.792 (4)	−0.5304 (17)	0.057 (3)	0.108 (3)
C17B	0.085 (4)	0.850 (4)	−0.4865 (18)	0.063 (3)	0.108 (3)
O4	0.3419 (2)	0.61974 (16)	0.14423 (16)	0.0398 (4)	
H4A	0.249 (3)	0.637 (3)	0.099 (3)	0.060*	
H4B	0.348 (4)	0.696 (2)	0.145 (3)	0.060*	
N1	0.4646 (2)	0.31767 (17)	0.11651 (16)	0.0266 (4)	
C1	0.4004 (2)	0.2356 (2)	0.0716 (2)	0.0268 (4)	
H1	0.367084	0.267018	−0.015287	0.032*	
C2	0.3811 (3)	0.1067 (2)	0.1480 (2)	0.0290 (4)	
C3	0.4287 (3)	0.0613 (2)	0.2745 (2)	0.0396 (5)	
H3	0.417793	−0.026974	0.328735	0.048*	
C4	0.4926 (3)	0.1471 (2)	0.3205 (2)	0.0424 (6)	
H4	0.525059	0.119055	0.407196	0.051*	
C5	0.5084 (3)	0.2742 (2)	0.2385 (2)	0.0344 (5)	
H5	0.552288	0.332680	0.270620	0.041*	

### Atomic displacement parameters (Å^2^)

**Table:**

	*U*^11^	*U*^22^	*U*^33^	*U*^12^	*U*^13^	*U*^23^
Cu1	0.0231 (2)	0.02040 (19)	0.0430 (2)	−0.00928 (14)	0.00053 (15)	−0.00916 (15)
O1	0.0402 (17)	0.0193 (12)	0.0609 (18)	−0.0079 (10)	0.0007 (12)	−0.0177 (12)
N2	0.0295 (11)	0.0248 (13)	0.0500 (17)	−0.0128 (10)	0.0012 (11)	−0.0152 (12)
C6	0.0254 (16)	0.0212 (13)	0.044 (2)	−0.0112 (11)	0.0099 (12)	−0.0135 (14)
C7	0.0341 (12)	0.0339 (16)	0.0435 (18)	−0.0179 (11)	0.0028 (12)	−0.0162 (14)
O1B	0.0402 (17)	0.0193 (12)	0.0609 (18)	−0.0079 (10)	0.0007 (12)	−0.0177 (12)
N2B	0.030 (4)	0.024 (4)	0.045 (5)	−0.013 (4)	0.004 (4)	−0.017 (4)
C6B	0.027 (5)	0.027 (5)	0.045 (5)	−0.010 (5)	0.004 (5)	−0.016 (5)
C7B	0.0341 (12)	0.0339 (16)	0.0435 (18)	−0.0179 (11)	0.0028 (12)	−0.0162 (14)
O2	0.0191 (17)	0.0266 (9)	0.0428 (11)	−0.0075 (14)	−0.0012 (13)	−0.0106 (8)
O3	0.0318 (9)	0.0459 (11)	0.0487 (18)	−0.0020 (8)	−0.0003 (14)	−0.0235 (15)
O5	0.122 (3)	0.141 (4)	0.084 (2)	−0.089 (3)	0.032 (2)	−0.002 (2)
O6	0.0442 (12)	0.0922 (18)	0.0550 (13)	−0.0360 (12)	0.0108 (10)	−0.0180 (12)
C8	0.0248 (14)	0.0230 (18)	0.0409 (16)	−0.0083 (11)	−0.0007 (13)	−0.0065 (12)
C9	0.0254 (13)	0.0390 (14)	0.0412 (14)	−0.0061 (11)	0.0007 (10)	−0.0147 (12)
C10	0.067 (2)	0.075 (2)	0.0416 (18)	−0.0075 (19)	0.0022 (16)	−0.0096 (17)
C11	0.060 (2)	0.075 (3)	0.050 (2)	0.0060 (19)	−0.0126 (18)	−0.0309 (18)
C12	0.0530 (18)	0.071 (2)	0.062 (2)	−0.0139 (16)	−0.0056 (16)	−0.0364 (17)
C13	0.0494 (16)	0.0487 (17)	0.0488 (16)	−0.0130 (13)	0.0008 (13)	−0.0224 (14)
C14	0.0356 (13)	0.0423 (15)	0.0388 (14)	−0.0042 (11)	0.0050 (11)	−0.0175 (12)
C15	0.0290 (12)	0.0466 (14)	0.0432 (15)	−0.0072 (11)	0.0031 (11)	−0.0160 (12)
C16	0.0511 (16)	0.064 (2)	0.0408 (15)	−0.0157 (16)	0.0071 (13)	−0.0146 (14)
C17	0.069 (2)	0.087 (3)	0.056 (2)	−0.036 (2)	0.0193 (17)	−0.0121 (18)
O2B	0.0191 (17)	0.0266 (9)	0.0428 (11)	−0.0075 (14)	−0.0012 (13)	−0.0106 (8)
O3B	0.0318 (9)	0.0459 (11)	0.0487 (18)	−0.0020 (8)	−0.0003 (14)	−0.0235 (15)
O5B	0.100 (10)	0.093 (11)	0.076 (10)	−0.041 (10)	0.019 (9)	−0.017 (9)
O6B	0.050 (4)	0.073 (5)	0.052 (4)	−0.026 (4)	0.010 (4)	−0.012 (4)
C8B	0.026 (5)	0.032 (5)	0.041 (4)	−0.010 (4)	0.003 (4)	−0.013 (4)
C9B	0.027 (5)	0.038 (5)	0.041 (5)	−0.009 (4)	−0.001 (4)	−0.015 (4)
C10B	0.059 (6)	0.073 (6)	0.044 (5)	−0.014 (5)	0.002 (5)	−0.014 (5)
C11B	0.060 (6)	0.071 (6)	0.047 (6)	−0.009 (5)	−0.005 (5)	−0.022 (5)
C12B	0.055 (4)	0.065 (5)	0.048 (5)	−0.011 (4)	−0.005 (4)	−0.024 (4)
C13B	0.051 (4)	0.062 (4)	0.048 (4)	−0.010 (4)	0.000 (4)	−0.024 (4)
C14B	0.043 (4)	0.056 (4)	0.042 (4)	−0.013 (4)	0.001 (4)	−0.020 (4)
C15B	0.036 (4)	0.054 (4)	0.044 (4)	−0.014 (4)	0.003 (4)	−0.017 (4)
C16B	0.056 (4)	0.070 (4)	0.044 (4)	−0.021 (4)	0.005 (4)	−0.015 (4)
C17B	0.057 (5)	0.081 (5)	0.052 (5)	−0.028 (5)	0.011 (4)	−0.016 (5)
O4	0.0401 (9)	0.0262 (8)	0.0560 (11)	−0.0102 (7)	0.0014 (8)	−0.0168 (8)
N1	0.0218 (8)	0.0217 (8)	0.0405 (10)	−0.0087 (6)	0.0036 (7)	−0.0140 (7)
C1	0.0209 (9)	0.0248 (10)	0.0379 (11)	−0.0067 (8)	0.0034 (8)	−0.0147 (9)
C2	0.0225 (9)	0.0222 (9)	0.0452 (12)	−0.0083 (8)	0.0077 (9)	−0.0142 (9)
C3	0.0444 (13)	0.0270 (11)	0.0473 (14)	−0.0164 (10)	0.0033 (11)	−0.0073 (10)
C4	0.0539 (15)	0.0396 (13)	0.0367 (13)	−0.0211 (11)	−0.0009 (11)	−0.0095 (10)
C5	0.0354 (12)	0.0338 (11)	0.0410 (13)	−0.0163 (9)	0.0023 (9)	−0.0163 (10)

### Geometric parameters (Å, º)

**Table:**

Cu1—O2^i^	2.008 (3)	C13—C14	1.381 (4)
Cu1—O2	2.008 (3)	C14—C15	1.506 (4)
Cu1—O4^i^	2.4790 (17)	C14—C16	1.369 (4)
Cu1—O4	2.4790 (17)	C15—H15	1.0000
Cu1—N1	2.0146 (16)	C16—C17	1.473 (5)
Cu1—N1^i^	2.0146 (16)	O2B—C8B	1.270 (18)
O1—C6	1.228 (3)	O3B—C8B	1.260 (18)
N2—H2	0.849 (17)	O5B—C17B	1.186 (17)
N2—C6	1.338 (4)	O6B—C15B	1.460 (16)
N2—C7	1.454 (3)	O6B—C17B	1.374 (17)
C6—C2	1.510 (3)	C8B—C9B	1.543 (17)
C7—C7^ii^	1.518 (6)	C9B—H9BA	0.9900
C7—H7A	0.9900	C9B—H9BB	0.9900
C7—H7B	0.9900	C9B—C15B	1.494 (17)
O1B—C6B	1.206 (18)	C10B—H10B	0.9500
N2B—H2B	0.8800	C10B—C11B	1.368 (19)
N2B—C6B	1.334 (17)	C10B—C16B	1.397 (17)
N2B—C7B	1.478 (17)	C11B—H11B	0.9500
C6B—C2	1.505 (17)	C11B—C12B	1.39 (2)
C7B—C7B^ii^	1.48 (5)	C12B—H12B	0.9500
C7B—H7BA	0.9900	C12B—C13B	1.396 (18)
C7B—H7BB	0.9900	C13B—H13B	0.9500
O2—C8	1.268 (4)	C13B—C14B	1.397 (17)
O3—C8	1.250 (4)	C14B—C15B	1.516 (16)
O5—C17	1.197 (5)	C14B—C16B	1.356 (17)
O6—C15	1.450 (3)	C15B—H15B	1.0000
O6—C17	1.368 (4)	C16B—C17B	1.468 (17)
C8—C9	1.516 (4)	O4—H4A	0.855 (18)
C9—H9A	0.9900	O4—H4B	0.814 (18)
C9—H9B	0.9900	N1—C1	1.343 (2)
C9—C15	1.505 (4)	N1—C5	1.329 (3)
C10—H10	0.9500	C1—H1	0.9500
C10—C11	1.363 (6)	C1—C2	1.387 (3)
C10—C16	1.399 (5)	C2—C3	1.381 (3)
C11—H11	0.9500	C3—H3	0.9500
C11—C12	1.377 (6)	C3—C4	1.384 (3)
C12—H12	0.9500	C4—H4	0.9500
C12—C13	1.388 (4)	C4—C5	1.382 (3)
C13—H13	0.9500	C5—H5	0.9500
			
O2^i^—Cu1—O2	180.0	O6—C15—C14	103.8 (2)
O2—Cu1—O4	94.84 (13)	O6—C15—H15	109.9
O2^i^—Cu1—O4^i^	94.84 (13)	C9—C15—C14	113.1 (2)
O2—Cu1—O4^i^	85.16 (13)	C9—C15—H15	109.9
O2^i^—Cu1—O4	85.16 (13)	C14—C15—H15	109.9
O2^i^—Cu1—N1^i^	90.32 (15)	C10—C16—C17	129.9 (3)
O2—Cu1—N1^i^	89.68 (15)	C14—C16—C10	121.5 (4)
O2^i^—Cu1—N1	89.68 (15)	C14—C16—C17	108.6 (3)
O2—Cu1—N1	90.32 (15)	O5—C17—O6	121.0 (4)
O2B^i^—Cu1—O2B	180.0 (11)	O5—C17—C16	131.3 (4)
O2B^i^—Cu1—O4	81.9 (14)	O6—C17—C16	107.7 (3)
O2B—Cu1—O4	98.1 (14)	C8B—O2B—Cu1	139 (3)
O2B—Cu1—N1	91.3 (16)	C17B—O6B—C15B	110.4 (13)
O2B^i^—Cu1—N1	88.7 (16)	O2B—C8B—C9B	118 (2)
O2B^i^—Cu1—N1^i^	91.3 (16)	O3B—C8B—O2B	121 (3)
O2B—Cu1—N1^i^	88.7 (16)	O3B—C8B—C9B	119 (2)
O4^i^—Cu1—O4	180.0	C8B—C9B—H9BA	108.5
N1^i^—Cu1—O4^i^	88.64 (6)	C8B—C9B—H9BB	108.5
N1—Cu1—O4^i^	91.36 (6)	H9BA—C9B—H9BB	107.5
N1^i^—Cu1—O4	91.36 (6)	C15B—C9B—C8B	115.2 (19)
N1—Cu1—O4	88.64 (6)	C15B—C9B—H9BA	108.5
N1^i^—Cu1—N1	180.00 (10)	C15B—C9B—H9BB	108.5
C6—N2—H2	117.4 (19)	C11B—C10B—H10B	121.3
C6—N2—C7	122.8 (2)	C11B—C10B—C16B	117 (2)
C7—N2—H2	116.2 (19)	C16B—C10B—H10B	121.3
O1—C6—N2	124.5 (2)	C10B—C11B—H11B	118.6
O1—C6—C2	120.0 (2)	C10B—C11B—C12B	123 (2)
N2—C6—C2	115.5 (2)	C12B—C11B—H11B	118.6
N2—C7—C7^ii^	111.6 (3)	C11B—C12B—H12B	120.1
N2—C7—H7A	109.3	C11B—C12B—C13B	120 (2)
N2—C7—H7B	109.3	C13B—C12B—H12B	120.1
C7^ii^—C7—H7A	109.3	C12B—C13B—H13B	121.9
C7^ii^—C7—H7B	109.3	C12B—C13B—C14B	116.1 (18)
H7A—C7—H7B	108.0	C14B—C13B—H13B	121.9
C6B—N2B—H2B	122.6	C13B—C14B—C15B	129.4 (15)
C6B—N2B—C7B	114.9 (16)	C16B—C14B—C13B	123.6 (15)
C7B—N2B—H2B	122.6	C16B—C14B—C15B	106.9 (13)
O1B—C6B—N2B	129 (2)	O6B—C15B—C9B	108.6 (15)
O1B—C6B—C2	126 (2)	O6B—C15B—C14B	104.4 (12)
N2B—C6B—C2	104.8 (15)	O6B—C15B—H15B	109.8
N2B—C7B—H7BA	110.3	C9B—C15B—C14B	114.2 (16)
N2B—C7B—H7BB	110.3	C9B—C15B—H15B	109.8
C7B^ii^—C7B—H7BA	110.3	C14B—C15B—H15B	109.8
C7B^ii^—C7B—H7BB	110.3	C10B—C16B—C17B	128.5 (18)
H7BA—C7B—H7BB	108.6	C14B—C16B—C10B	119.8 (17)
C8—O2—Cu1	127.6 (3)	C14B—C16B—C17B	111.1 (14)
C17—O6—C15	111.1 (2)	O5B—C17B—O6B	120 (2)
O2—C8—C9	116.7 (3)	O5B—C17B—C16B	133 (2)
O3—C8—O2	125.9 (3)	O6B—C17B—C16B	106.7 (14)
O3—C8—C9	117.4 (3)	Cu1—O4—H4A	86 (2)
C8—C9—H9A	108.2	Cu1—O4—H4B	124 (2)
C8—C9—H9B	108.2	H4A—O4—H4B	106 (3)
H9A—C9—H9B	107.4	C1—N1—Cu1	120.45 (14)
C15—C9—C8	116.4 (2)	C5—N1—Cu1	120.88 (13)
C15—C9—H9A	108.2	C5—N1—C1	118.64 (18)
C15—C9—H9B	108.2	N1—C1—H1	118.9
C11—C10—H10	121.1	N1—C1—C2	122.12 (19)
C11—C10—C16	117.8 (4)	C2—C1—H1	118.9
C16—C10—H10	121.1	C1—C2—C6	119.5 (2)
C10—C11—H11	119.6	C1—C2—C6B	131.6 (16)
C10—C11—C12	120.8 (3)	C3—C2—C6	121.5 (2)
C12—C11—H11	119.6	C3—C2—C6B	109.2 (15)
C11—C12—H12	119.1	C3—C2—C1	118.97 (19)
C11—C12—C13	121.7 (3)	C2—C3—H3	120.7
C13—C12—H12	119.1	C2—C3—C4	118.7 (2)
C12—C13—H13	121.2	C4—C3—H3	120.7
C14—C13—C12	117.6 (3)	C3—C4—H4	120.5
C14—C13—H13	121.2	C5—C4—C3	119.1 (2)
C13—C14—C15	130.6 (3)	C5—C4—H4	120.5
C16—C14—C13	120.6 (3)	N1—C5—C4	122.5 (2)
C16—C14—C15	108.7 (3)	N1—C5—H5	118.7
O6—C15—C9	110.0 (2)	C4—C5—H5	118.7
			
Cu1—O2—C8—O3	−14.2 (8)	C16—C10—C11—C12	1.5 (5)
Cu1—O2—C8—C9	168.7 (3)	C16—C14—C15—O6	−2.4 (3)
Cu1—O2B—C8B—O3B	11 (10)	C16—C14—C15—C9	116.8 (3)
Cu1—O2B—C8B—C9B	178 (4)	C17—O6—C15—C9	−119.8 (3)
Cu1—N1—C1—C2	177.22 (14)	C17—O6—C15—C14	1.5 (3)
Cu1—N1—C5—C4	−177.37 (17)	O2B—C8B—C9B—C15B	−118 (4)
O1—C6—C2—C1	136.2 (4)	O3B—C8B—C9B—C15B	49 (5)
O1—C6—C2—C3	−45.1 (5)	C8B—C9B—C15B—O6B	54 (3)
N2—C6—C2—C1	−43.4 (4)	C8B—C9B—C15B—C14B	170 (2)
N2—C6—C2—C3	135.2 (3)	C10B—C11B—C12B—C13B	5 (6)
C6—N2—C7—C7^ii^	83.9 (5)	C10B—C16B—C17B—O5B	−3 (8)
C6—C2—C3—C4	−178.1 (3)	C10B—C16B—C17B—O6B	175 (4)
C7—N2—C6—O1	5.3 (6)	C11B—C10B—C16B—C14B	−4 (5)
C7—N2—C6—C2	−175.1 (3)	C11B—C10B—C16B—C17B	−174 (4)
O1B—C6B—C2—C1	121 (4)	C11B—C12B—C13B—C14B	−2 (5)
O1B—C6B—C2—C3	−53 (5)	C12B—C13B—C14B—C15B	173 (3)
N2B—C6B—C2—C1	−59 (4)	C12B—C13B—C14B—C16B	−4 (5)
N2B—C6B—C2—C3	127 (2)	C13B—C14B—C15B—O6B	177 (3)
C6B—N2B—C7B—C7B^ii^	169 (4)	C13B—C14B—C15B—C9B	59 (4)
C6B—C2—C3—C4	175.3 (15)	C13B—C14B—C16B—C10B	7 (5)
C7B—N2B—C6B—O1B	−1 (7)	C13B—C14B—C16B—C17B	179 (3)
C7B—N2B—C6B—C2	179 (2)	C14B—C16B—C17B—O5B	−174 (4)
O2—C8—C9—C15	−16.2 (6)	C14B—C16B—C17B—O6B	4 (4)
O3—C8—C9—C15	166.5 (4)	C15B—O6B—C17B—O5B	171 (3)
C8—C9—C15—O6	−67.6 (3)	C15B—O6B—C17B—C16B	−7 (4)
C8—C9—C15—C14	176.8 (3)	C15B—C14B—C16B—C10B	−171 (3)
C10—C11—C12—C13	−1.0 (5)	C15B—C14B—C16B—C17B	1 (4)
C10—C16—C17—O5	−2.6 (8)	C16B—C10B—C11B—C12B	−2 (6)
C10—C16—C17—O6	179.2 (4)	C16B—C14B—C15B—O6B	−5 (3)
C11—C10—C16—C14	−0.8 (6)	C16B—C14B—C15B—C9B	−123 (3)
C11—C10—C16—C17	178.4 (4)	C17B—O6B—C15B—C9B	130 (2)
C11—C12—C13—C14	−0.4 (5)	C17B—O6B—C15B—C14B	8 (3)
C12—C13—C14—C15	178.2 (3)	O4—Cu1—O2B—C8B	1 (7)
C12—C13—C14—C16	1.1 (4)	O4^i^—Cu1—O2B—C8B	−179 (7)
C13—C14—C15—O6	−179.8 (3)	N1—Cu1—O2B—C8B	90 (7)
C13—C14—C15—C9	−60.6 (4)	N1^i^—Cu1—O2B—C8B	−90 (7)
C13—C14—C16—C10	−0.5 (5)	N1—C1—C2—C6	179.0 (2)
C13—C14—C16—C17	−179.9 (3)	N1—C1—C2—C6B	−173.1 (18)
C14—C16—C17—O5	176.6 (5)	N1—C1—C2—C3	0.3 (3)
C14—C16—C17—O6	−1.5 (4)	C1—N1—C5—C4	0.9 (3)
C15—O6—C17—O5	−178.5 (4)	C1—C2—C3—C4	0.5 (3)
C15—O6—C17—C16	−0.1 (4)	C2—C3—C4—C5	−0.7 (4)
C15—C14—C16—C10	−178.2 (3)	C3—C4—C5—N1	0.0 (4)
C15—C14—C16—C17	2.4 (4)	C5—N1—C1—C2	−1.0 (3)

Symmetry codes: (i) −*x*+1, −*y*+1, −*z*; (ii) −*x*, −*y*, −*z*.

### Hydrogen-bond geometry (Å, º)

**Table:**

*D*—H···*A*	*D*—H	H···*A*	*D*···*A*	*D*—H···*A*
N2—H2···O3^iii^	0.85 (2)	1.98 (2)	2.801 (4)	163 (3)
O4—H4*A*···O3	0.86 (2)	1.83 (2)	2.674 (4)	170 (3)
O4—H4*B*···O1^iv^	0.81 (2)	2.05 (2)	2.860 (3)	171 (3)
C1—H1···O2	0.95	2.52	2.987 (5)	111

Symmetry codes: (iii) −*x*, −*y*+1, −*z*; (iv) *x*, *y*+1, *z*.
